# Supplementary material for: Molecular architecture of softwood revealed by solid-state NMR
Source: Nat Commun. 2019 Oct 31;10:4978. doi: 10.1038/s41467-019-12979-9 (PMC6823442; doi:10.1038/s41467-019-12979-9)
Supplement: Supplementary file 1 — Supplementary Information [file 41467_2019_12979_MOESM1_ESM.pdf]

Supplementary Information

Molecular architecture of softwood revealed by solid-state NMR

Oliver M. Terrett[1], Jan J. Lyczakowski[1,2], Li Yu[1,2], Dinu Iuga[3], Trent W. Franks[3], Steven P. Brown[3], Ray Dupree[3]* and Paul Dupree[1,2]*

1. Department of Biochemistry, University of Cambridge, Hopkins Building, The Downing Site, Tennis Court Road, Cambridge CB2 1QW, UK
2. Natural Material Innovation Centre, University of Cambridge, 1 Scroope Terrace, Cambridge, CB2 1PX, UK
3. Department of Physics, University of Warwick, Coventry, CV4 7AL, UK

*Joint corresponding authors

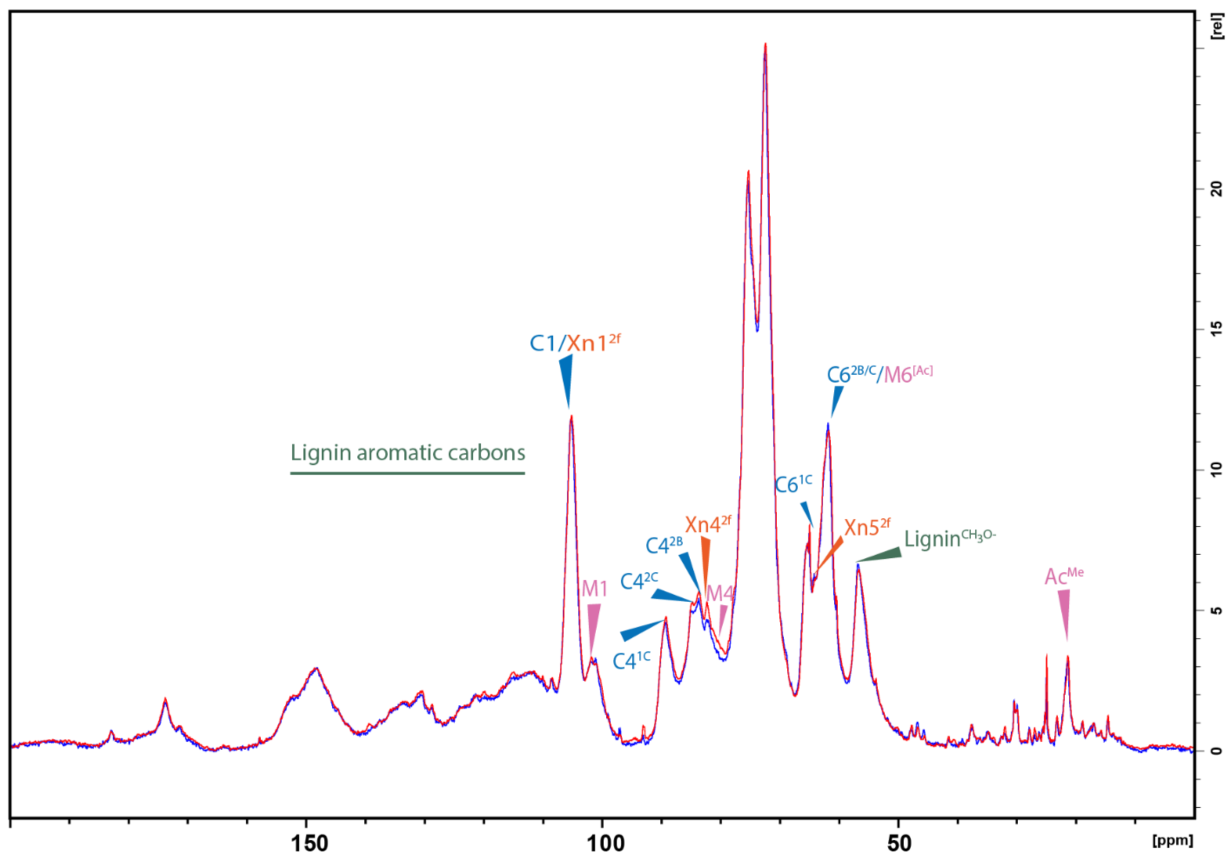

Supplementary Figure 1 One-dimensional 20 s DP spectra of spruce before and after the experiments. 20 s DP spectra are shown, in blue is the spectrum taken before multidimensional experiments, and in blue is the spectrum taken 1 week later after multi-dimensional experiments. There are no significant changes, and no decomposition has occurred. Some polysaccharide carbons have been annotated on the spectra.

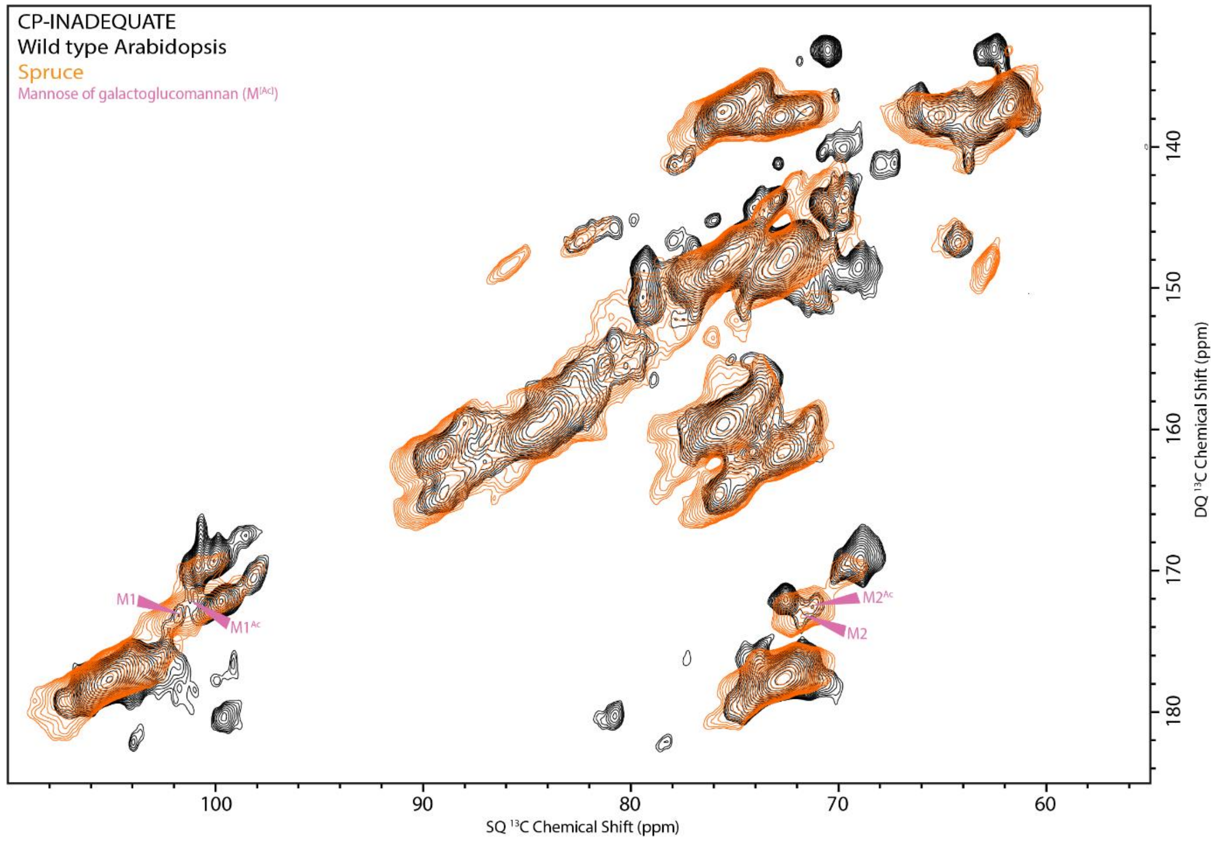

Supplementary Figure 2 Comparison of Arabidopsis and spruce GGM chemical shifts. Overlaid refocussed ^{13}C CP-INADEQUATE MAS NMR spectra of wild type Arabidopsis and spruce, the wild type Arabidopsis spectrum was previously published in (Grantham et al ., 2017)[1]. The peaks corresponding to $M1^{[Ac]}$ and $M2^{[Ac]}$ are labelled. The superscript Ac indicates that the residue is acetylated.

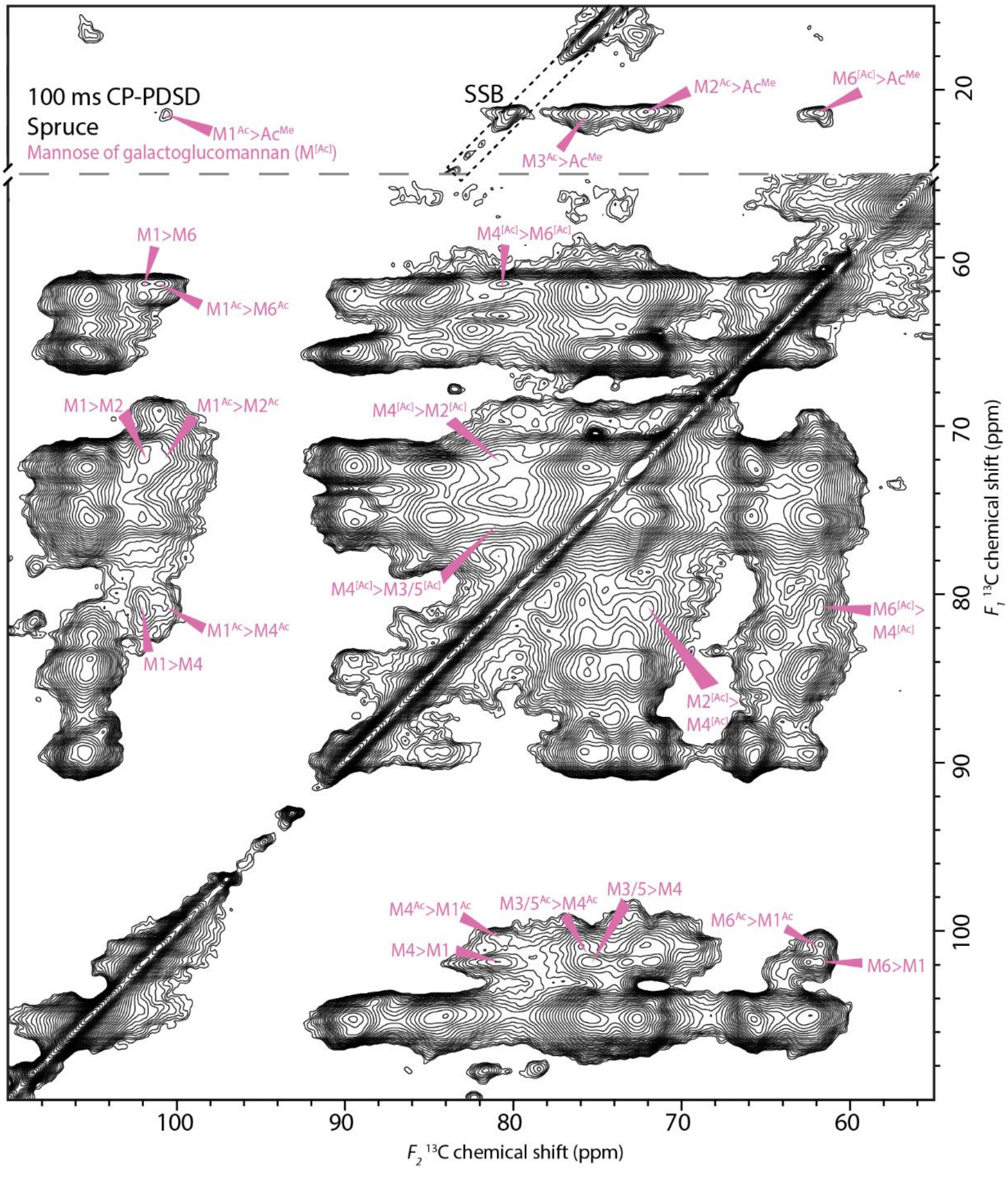

Supplementary Figure 3 Assigning the acetylated mannose residue of GGM. The acetate methyl (Ac^{Me}) and carbohydrate regions of a 100 ms mixing time ^{13}C CP-PDSD MAS NMR spectra of spruce is shown. The internal cross-peaks within the two GGM mannose residues are labelled. The superscript Ac denotes the acetylated mannose residue. Spinning side bands are surrounded by black dotted lines and are marked SSB.

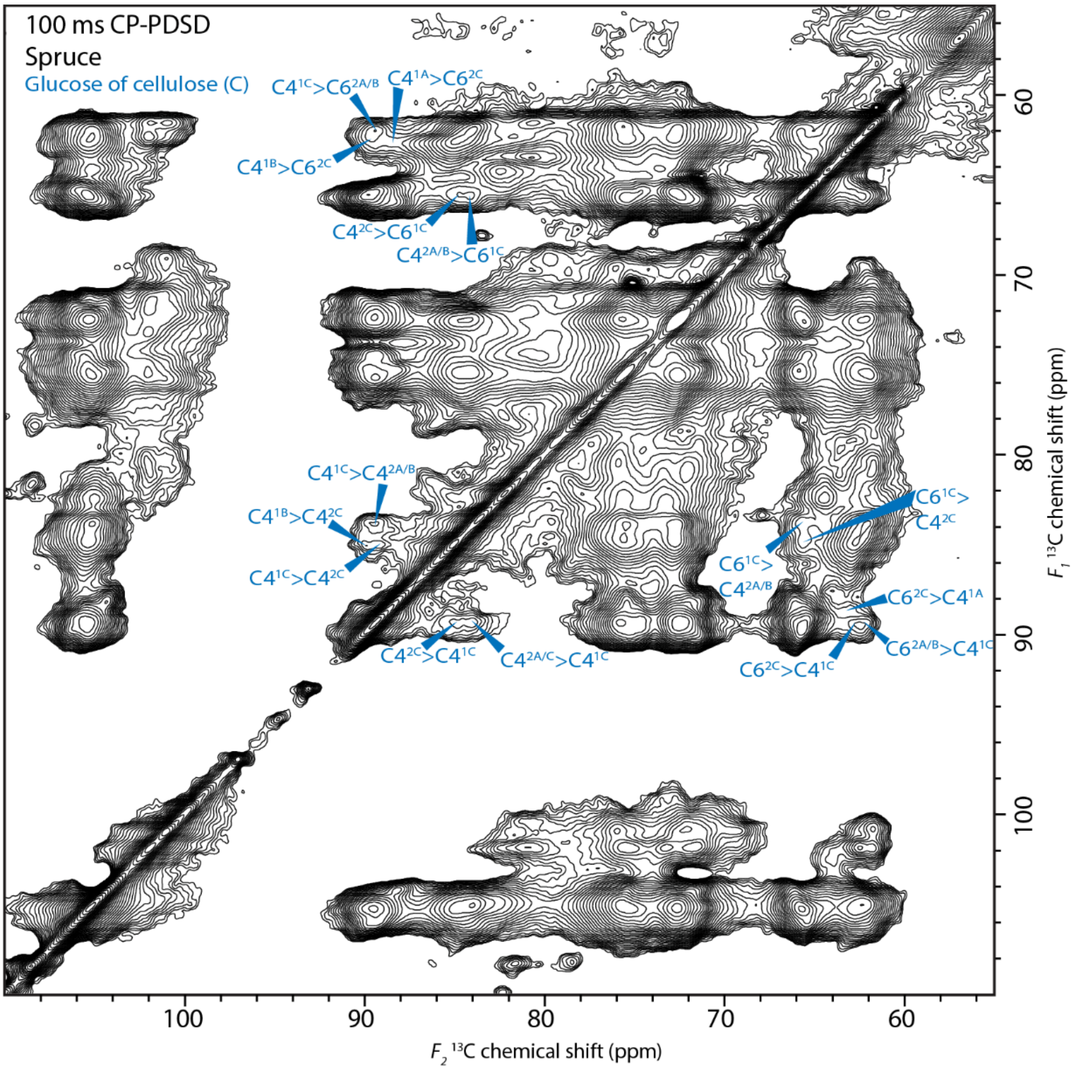

Supplementary Figure 4 Different domains of cellulose are in very close proximity. The carbohydrate region of a 100 ms mixing time ^{13}C CP-PDSD MAS NMR spectrum of spruce is shown. The cross-peaks between the sub-domains of cellulose are labelled.

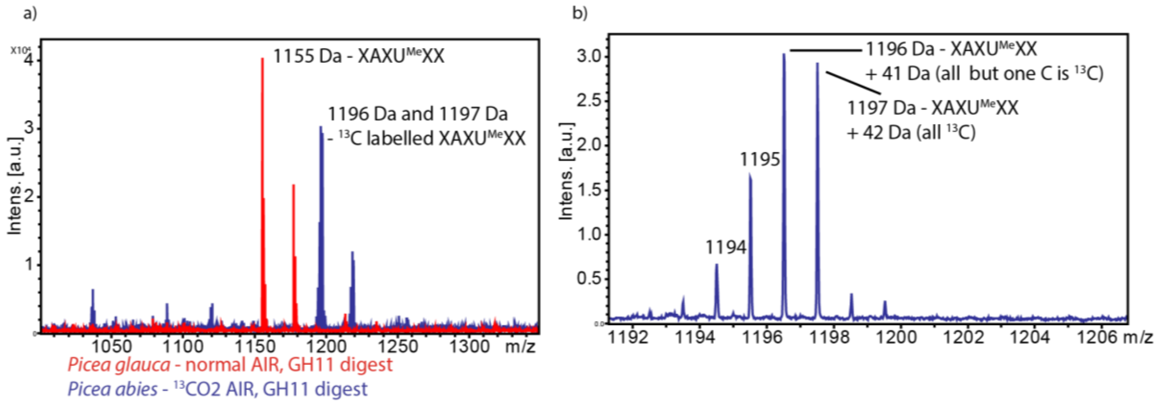

Supplementary Figure 5 MS analysis of ^{13}C enrichment of spruce wood sample. a) A GH11 xylanase digest was performed on alkali extracted xylan from *Picea glauca* grown naturally (red spectrum) and *Picea abies* purchased from Isolife (blue spectrum). The oligosaccharide products are labelled after the conventions in[2]. b) The degree of enrichment was calculated from the volume integration of XAXUXX $[M+Na^{+}]^{+}$ peaks with different masses, and is over 97%.

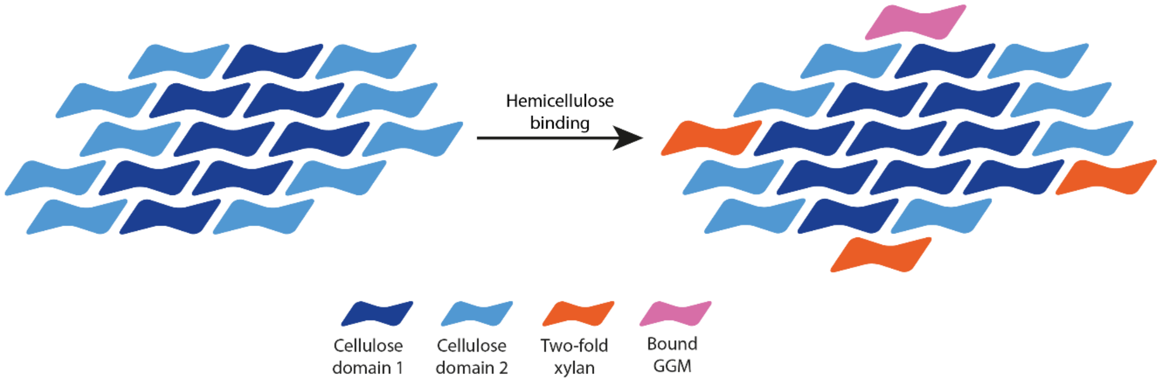

Supplementary Figure 6 Proposed mechanism for hydrophilic face binding converting surface domain 2 chains into domain 1 chains. Initially, an 18 chain microfibril is shown with nearly all domain 1 (C4 ≈89 ppm) chains in the interior, and domain 2 (C4 ≈84 ppm) chains on the surface. If xylan binds to the hydrophilic face, the carbon 2 and 3 could hydrogen bond to the carbon 6 of the surface glucan chains and fix the hydroxymethyl conformation, converting the surface chains from domain 2 to domain 1. However, this xylan driven domain conversion leads to a change of domain ratio from preferred 8:10 (domain 1: domain 2) to 10:8, when two xylan chains are bound. If xylan and GGM bind to the hydrophobic faces of cellulose, they would not hydrogen bond to the carbon 6 and may not affect the carbon 6 hydroxymethyl conformation.

Supplementary Table 1 Non-cellulosic monosaccharide composition analysis of ^{13}C spruce sample**.**

Monosaccharide	mol %
Fucose	0.9
Arabinose	8.3
Galactose	9.6
Glucose	21.0
Xylose	21.2
Mannose	36.3
Galacturonic Acid	1.4
Glucuronic Acid	1.4

The molar percentage of non-cellulosic monosaccharides from TFA hydrolysis of ^{13}C spruce AIR is shown.

Supplementary Table 2 Chemical shift assignments of spruce cell wall components.

Polysaccharide or moiety		Carbon number and chemical shift (ppm)						Notes, references
Name	Type, moiety, functionality	1	2	3	4	5	6	
Cellulose	Domain 1^A	104.3	-	-	88.3	71.7	66.0	Assignment presented in this work
	Domain 1^B	106.0	71.6	75.3	90.0	71.7	65.8	
	Domain 1^C	105.4	72.4	75.9	89.5	72.8	65.5	
	Domain 2^A	105.3	72.6	75.4	84.1	74.3	62.2	
	Domain 2^B	105.2	-	74.2	83.7	75.6	61.9	
	Domain 2^C	105.3	72.7	75.6	84.5	75.2	62.6	
Xylan	2-fold screw xylose (Xn^{2f})	105.2	72.3	75.2	82.4	64.3	n/a	Carbon 1 to 3 values after (Simmons et al., 2016[3])
	3-fold screw xylose (Xn^{3f})	102.6	73.7	74.7	77.7	63.9	n/a	
Galactoglucomannan (GGM)	GGM (M)	101.9	72.0	-	80.4	75.8	61.6	Assignment presented in this work
	Acetylated GGM (M^{Ac})	100.9	71.9	75.9	80.4	75.8	61.6	
	GGM (M)	101.2	71.1-71.5	72.6-73.8	67.6-78.5	76.1-77.5	61.5-61.9	Data from solution state NMR for spruce mannan. Cited after (Hannuksela et al., 2004[4])
	Acetylated GGM (M^{Ac})	99.8-100.6	69.2-72.7	71.2-74.4	74.2-78.5	76.1-76.3	-	
	Mannan I (M^I)	101.7-101.9	70.2	72.5	80.4-81.3	76.2	61.9-62.3	Mannan I after (Daud et al., 1992[5]; Heux et al., 2005[6]).
Xylan arabinose or AG	t-Ara^1	108.6	82.1	78.3	86.1	62.8	n/a	Assignment presented in this work
	t-Ara^2	110.2	82.5	77.8	85.6	62.8	n/a	
AG/GGM galactose or pectin galacturonic acid	Gal/GalA	101.1	69.8	-	-	-	-	Assignment presented in this work

For lignin following assignments were used: CH_3O- (56.5 ppm), G_3 (148.0-150.0 ppm), $G_{aromatic}$ (128.0-130.0 ppm).

The ^{13}C chemical shift assignments are collated. For comparison to the *in muro* spruce GGM, chemical shifts from the solution-state NMR of spruce GGM and solid-state NMR of semicrystalline mannan I are shown.

Supplementary Table 3 ^{13}C spin lattice relaxation times, T_1, at 176.0 MHz Larmor frequency of select chemical shifts from ^{13}C labelled spruce.

	Chemical shift (ppm)	T_1 long (s)	T_1 short (s)	Fraction short T_1
Ac^{Me}	21.4	4.3 ± 0.53	0.72 ± 0.16	0.33
Lignin CH_3O	56.5	3.1 ± 0.21	--	--
$M6^{[Ac]}$	61.7	4.8 ± 0.24	0.27 ± 0.04	0.23
$C6^{2C}$	62.4	5.0 ± 0.16	0.26 ± 0.05	0.12
$Xn5^{2f}$	64	4.8 ± 0.19	0.32 ± 0.07	0.14
$C6^{1A}$	64.9	5.4 ± 0.08	0.25 ± 0.07	0.05
$C6^{1C}$	65.5	5.8 ± 0.03	--	--
	72.3	5.8 ± 0.07	0.59 ± 0.05	0.10
	74.9	5.8 ± 0.05	0.68 ± 0.05	0.08
	75.4	5.8 ± 0.06	0.66 ± 0.05	0.09
$Xn4^{2f}$	82.2	5.4 ± 0.12	0.68 ± 0.07	0.16
$C4^{2B}$	83.7	5.5 ± 0.14	0.6 ± 0.12	0.09
$C4^{2C}$	84.6	5.7 ± 0.12	0.66 ± 0.12	0.10
$C4^{1C}$	89.1	6.3 ± 0.10	--	--
$M1^{Ac}$	100.7	5.4 ± 0.19	0.79 ± 0.07	0.26
M1	101.7	5.4 ± 0.24	0.93 ± 0.15	0.18
$C1/Xn1^{2f}$	105.1	6.0 ± 0.04	--	--
$t\text{-}Ara1^{1}$	108.1	5.9 ± 0.7	0.71 ± 0.14	0.33
$G_{aromatic}$	128.5	4.4 ± 0.4	0.52 ± 0.15	0.23
$G_{aromatic}$	130.1	3.0 ± 0.3	--	--
G3	148.1	4.2 ± 0.08	--	--
G3	150.9	4.4 ± 0.1	--	--

For many shift values the relaxation is better described by two exponentials and the fraction of the short T_1 component is given.

References

1. Grantham, N. J. *et al.* An even pattern of xylan substitution is critical for interaction with cellulose in plant cell walls. *Nat. Plants* **3**, 859 (2017).
2. Fauré, R. *et al.* A Brief and Informationally Rich Naming System for Oligosaccharide Motifs of Heteroxylans Found in Plant Cell Walls*. *Aust. J. Chem.* **62**, 533–537 (2009).
3. Simmons, T. J. *et al.* Folding of xylan onto cellulose fibrils in plant cell walls revealed by solid-state NMR. *Nat. Commun.* **7**, ncomms13902 (2016).
4. Hannuksela, T. & Hervé du Penhoat, C. NMR structural determination of dissolved O-acetylated galactoglucomannan isolated from spruce thermomechanical pulp. *Carbohydr. Res.* **339**, 301–312 (2004).
5. Daud, M. J. & Jarvis, M. C. Mannan of oil palm kernel. *Phytochemistry* **31**, 463–464 (1992).
6. Heux, L., Hägglund, P., Putaux, J.-L. & Chanzy, H. Structural Aspects in Semicrystalline Samples of the Mannan II Family. *Biomacromolecules* **6**, 324–332 (2005).
